# Gendered Association Between Employment Status and Unmet Health Care Needs Among Middle-Aged Koreans

**DOI:** 10.1093/geroni/igaa057.729

**Published:** 2020-12-16

**Authors:** Si Young Song, Hey Jung Jun, Susanna Joo, Sun Ah Lee

**Affiliations:** Yonsei University, Seoul, Republic of Korea

## Abstract

Previous studies show that working people are less likely to experience unmet health care needs than non-workers. Also, employment and health are located in gendered social contexts. The present study aims to examine the moderating effect of gender on the association between employment status and unmet health care needs among middle-aged Koreans. We conducted logistic regression using the Korean Health Panel data (in 2016 and 2017; N=2,573, age range=45-64). Having experiences unmet health care needs in 2017 was the binary dependent variable. Employment status in 2016 was the binary independent variable and gender was the moderating variable. Age, education level, marital status, annual income, household type, type of medical security, disability, self-rated health, the number of chronic diseases, and stress level in 2016 were also in the analytic model based on the Andersen’s health behavioral model. The percentages of middle-aged people experiencing unmet health care needs were 18% for working men, 11% for non-working men, 13% for working women, and 16% for non-working women. The result showed there was significant moderating effect of gender (B= .72, p< .05). Specifically, working men were less likely to experience unmet health care needs than non-working men. On the contrary, there was not the significant difference in experiencing unmet health care needs between working and non-working women. It indicates that it is necessary to supplement medical services for especially for middle-aged men who are not employed because they might experience considerable amounts of unmet health care needs.

